# Identification of a novel splicing mutation and genotype–phenotype correlations in rare PLS3-related childhood-onset osteoporosis

**DOI:** 10.1186/s13023-022-02380-z

**Published:** 2022-06-25

**Authors:** Zhichong Wu, Zhenhua Feng, Xiufen Zhu, Zhicheng Dai, Kaixing Min, Yong Qiu, Long Yi, Leilei Xu, Zezhang Zhu

**Affiliations:** 1 Division of Spine Surgery, Department of Orthopedic Surgery, Nanjing Drum Tower Hospital, The Affiliated Hospital of Nanjing University Medical School, Nanjing, China; 2 Joint Scoliosis Research Center of The Chinese University of Hong Kong and Nanjing University, Nanjing & Hong Kong, China; 3 Osteoporosis and Metabolic Bone Disease Center, Department of Orthopedic Surgery, Nanjing Drum Tower Hospital, The Affiliated Hospital of Nanjing University Medical School, Nanjing, China; 4 Jiangsu Key Laboratory of Molecular Medicine, School of Medicine, Nanjing University, Nanjing, China

**Keywords:** PLS3 gene, Mutation, Phenotype, Primary osteoporosis, Scoliosis

## Abstract

**Background:**

X-linked early-onset osteoporosis, caused by mutations in plastin3 (PLS3), is an extremely rare disease characterized by low bone mineral density (BMD) and recurrent osteoporotic fractures. There is limited information on genetic and phenotypic spectrum, as well as genotype–phenotype correlations of the disease. Moreover, whether decreased PLS3 levels were also involved in osteoporosis among subjects without PLS3 pathogenic mutations remains unknown.

**Methods:**

Whole-exome sequencing and bidirectional Sanger sequencing were performed for screening and validation of pathogenic mutations. Serum biochemical parameters and clinical information of the subjects were retrospectively collected. ELISA and online datasets were utilized to investigate the association between PLS3 expression and BMD.

**Results:**

We identified a novel splicing mutation (c.892-2A > G) which led to the skipping of exon 9 in a family with X-linked early-onset osteoporosis. Scoliosis represents a potential new phenotype in the patients harboring PLS3 mutations, which may be corrected by brace treatment. Genotype–phenotype analysis reveals that there was no significant difference in BMD z-scores between different types of reported mutations including this study (*p* = 0.5). There is a marginally significant negative correlation between age and BMD z-score (*p* = 0.059, r = − 0.30). The conditions of osteoporosis in all patients were improved after bisphosphonates therapy, with mean BMD z-score increased from − 2.9 to − 0.57 (*p* < 0.0001). Serum PLS3 levels in adolescents and adults without PLS3 pathogenic mutations but representing osteoporosis were also evaluated, while no association was found between bone mineral density and PLS3 levels (*p* > 0.05).

**Conclusions:**

Our findings expanded the mutation and phenotype spectrum of the rare disease and highlights the importance of early diagnosis and early treatment with bisphosphonates. More reports of cases with PLS3 mutation and function studies of the gene are warranted to understand genotype–phenotype correlations.

**Supplementary Information:**

The online version contains supplementary material available at 10.1186/s13023-022-02380-z.

## Introduction

Although osteoporosis is commonly considered a disease of the elderly, it is increasingly recognized among children. Criteria for the diagnosis of childhood-onset osteoporosis were the presence of low BMD with at least one vertebral or low-trauma long bone fracture [1]. Childhood-onset osteoporosis was typically divided into primary childhood and secondary osteoporosis. Unlike secondary osteoporosis which is often a consequence of chronic disease or the medications used for treatment, primary osteoporosis is much rarer and results from pathogenic mutations disrupting the normal synthesis and turnover of bone or cartilage[2]. In 2013, van Dijk et al. firstly identified plastin3 (PLS3) gene as a monogenetic cause of childhood-onset osteoporosis using X-linked whole-exome sequencing in 5 unrelated families [3]. Subsequently, more novel mutation sites in PLS3 gene responsible for X-linked osteoporosis were reported by other researchers [4]. PLS3, located on chromosome Xq23, encodes protein plastin 3 which is ubiquitously expressed in solid tissues and considered to be involved in cytoskeleton remodeling [5]. Patients with PLS3 induced X-linked osteoporosis present early-onset osteoporosis and recurrent osteoporotic fractures, usually without characteristic extraskeletal manifestation of OI such as joint hyperlaxity, blue sclerae or discolored teeth [3]. Due to the X-linked inheritance, males with PLS3 mutations often present a more severe phenotype than females [3].

It is widely accepted that deficiency of PLS3 caused by pathogenic mutations will lead to early-onset osteoporosis. However, the genetic spectrum, phenotypic variability and treatment approaches of PLS3 induced osteoporosis are not fully understood as hampered by its extremely low incidence [4]. Moreover, the association between PLS3 levels and osteoporosis in individuals without pathogenic mutations in PLS3 needs to be determined.

In the present study, we identified a novel PLS3 splicing mutation in a patient with early-onset osteoporotic fracture and scoliosis. Clinical manifestation, as well as genetic alternation, was evaluated for the family of the proband. Furthermore, genotype–phenotype relationships in PLS3-related early-onset primary osteoporosis and the relationship between PLS3 levels and osteoporosis in subjects without PLS3 pathogenic mutations were investigated.

## Methods

### Evaluation of the family with X-linked early-onset osteoporosis

The proband is a 16-year-old boy of Chinese origin who came to the department of spine surgery because of scoliosis and childhood-onset osteoporosis. The proband, along with his parents and younger brother, were included in the study.

Serum biochemical parameters and clinical information were retrospectively obtained from the medical records of the patients. Bone mineral density (BMD) was measured using dual-energy X-ray absorptiometry, with lumbar spine and hip selected as the region of interest. Age- and gender- specific BMD Z-score was calculated based on normal reference data of BMD [6]. The standing anteroposterior and lateral spinal radiographs were performed for the proband and his brother.

### Genomic DNA analysis

Genomics DNA used for gene sequencing was extracted from peripheral blood by the standard procedure using DNA Extraction Kit (QIAGEN, Tokyo, Japan). Whole exome sequencing (WES) was performed for the proband. The genomic DNA was fragmented randomly to about 260 base pairs and captured using Roche 64 M whole exome capture kit to construct exome libraries which were then sequenced on Hiseq2600 (IIumina Inc, CA, USA). Reads alignment against the reference human genome (GRCh37/hg19) was performed using BWA software. Single nucleotide variants (SNVs) and small InDels (< 10 bp) were called by using SOAP SNP software and SAM tools Pileup software, respectively. The frequency of annotated variants in normal population was calculated based on public databases, including ESP6500 database, 1000 genome database, EXAC database and GnomAD database. The average sequencing depth of the target region is ≥ 180X, and the proportion of sites with the average depth of the target region > 20 × is > 95%. Sanger sequencing of the identified potential pathogenic variant by whole exome sequencing was performed for the proband and his family members[7].

### RNA transcript analysis by reverse transcription PCR (RT‐PCR)

Total RNA was extracted from peripheral leukocytes by Trizol method. The quality and concentration of the total RNA were assessed by measuring absorption values at 260 and 280 nm. By using PrimerScript RT reagent kit with gDNA Eraser (TaKaRa, Tokyo, Japan), total RNA was reversely transcribed to cDNA which was then amplified by using specific primers, the sequence of the primers was as follows: Forward 5′-TGGTGAGACTTTGGAGGAACTTA-3′, Reverse 5′-CAGTCAATATCCTGGTTCTCTGG-3′. The PCR products were separated by agarose gel electrophoresis and sequenced by the Sanger method.

### Genotype and phenotype analysis

Previously reported mutations in PLS3 were reviewed, with common variants and synonymous mutations (c.321 T > A) excluded from the analysis [3, 8]. As the phenomenon of X-inactivation existing in female heterozygous carriers of PLS3 mutations may bias the result, they were also excluded from the genotype–phenotype correlation analysis [3, 9]. Clinical information of male patients harboring PLS3 mutations was collected. Including this study, a total of 43 male patients from 26 families were included.

### Evaluation of PLS3 levels

Peripheral blood samples were collected into EDTA-containing tubes and centrifuged at 3000 g for 10 min at room temperature to obtain plasma, which was subsequently aliquoted and stored at − 80 ℃. Plasma PLS3 concentrations in 77 adolescents aged 14–17 years were evaluated by commercial enzyme-linked immunosorbent assay (ELISA) kit (Mlbio, Shanghai, China) according to recommended procedures. The expression information in adults with high or low bone mineral density (BMD), were downloaded from the NCBI gene expression omnibus (GEO) (https://www.ncbi.nlm.nih.gov/geo/). Three publicity available datasets, GSE7429 (40 patients), GSE56814 (73 patients) and GSE56815 (80 patients), were used for the analysis of PLS3 expression. No pathogenic mutation in PLS3 was found in the included adolescents indicated by WES. Because of the extremely low frequency of pathogenic mutation of PLS3 in the general population, even in individuals with primary osteoporosis [10], the adults were considered to have no pathogenic mutations.

### Statistical analysis

All statistical analysis was conducted by SPSS v19.0. Paired or unpaired Student t test was used to determine whether there is significant difference between two groups. The One-way ANOVA test was utilized to analyze any significant difference among the five different types of mutations. Correlations between continues variables were analyzed based on linear Pearson’s correlation coefficient. Graphics of protein structure were performed using PyMol Molecular Graphics System v2.5, while other data was graphed using Graphpad Prism v8.3. A *p*-value less than 0.05 is considered statistically significant.

## Results

### Subjects and clinical presentation

**Fig. 1:**
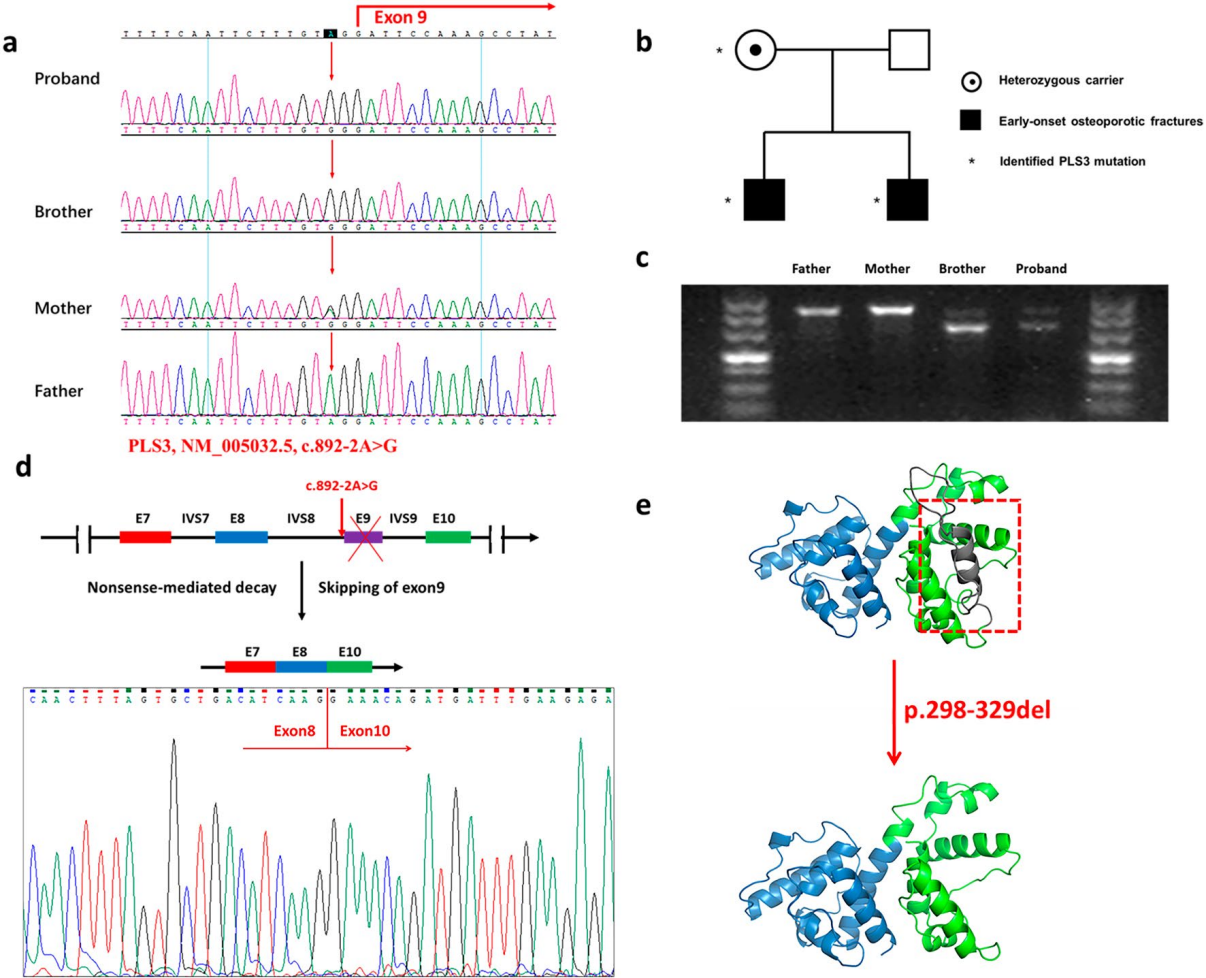
Pedigree and genetic analysis of the family. **a** Pedigree of the family showing an X-linked recessive inheritance. **b** Pathogenic mutation in PLS3 confirmed by sanger sequencing. **c** Gel image showing the RT-PCR products of PLS3 cDNA fragments. **d** Schematic representation of the alternation of the PLS3 gene and PLS3 protein (**e**) induced by the splicing mutation

The proband, of Chinese Han origin, came to our clinic center complaining of scoliosis and recurrent fractures. His younger brother visited the clinic due to low bone mineral density. Both brothers and their parents were included in the study (Fig. 1a). Adolescents aged between 14 and 17 years came to our clinical center and performed DXA examinations were included in the ELISA experiment.

**Fig. 2:**
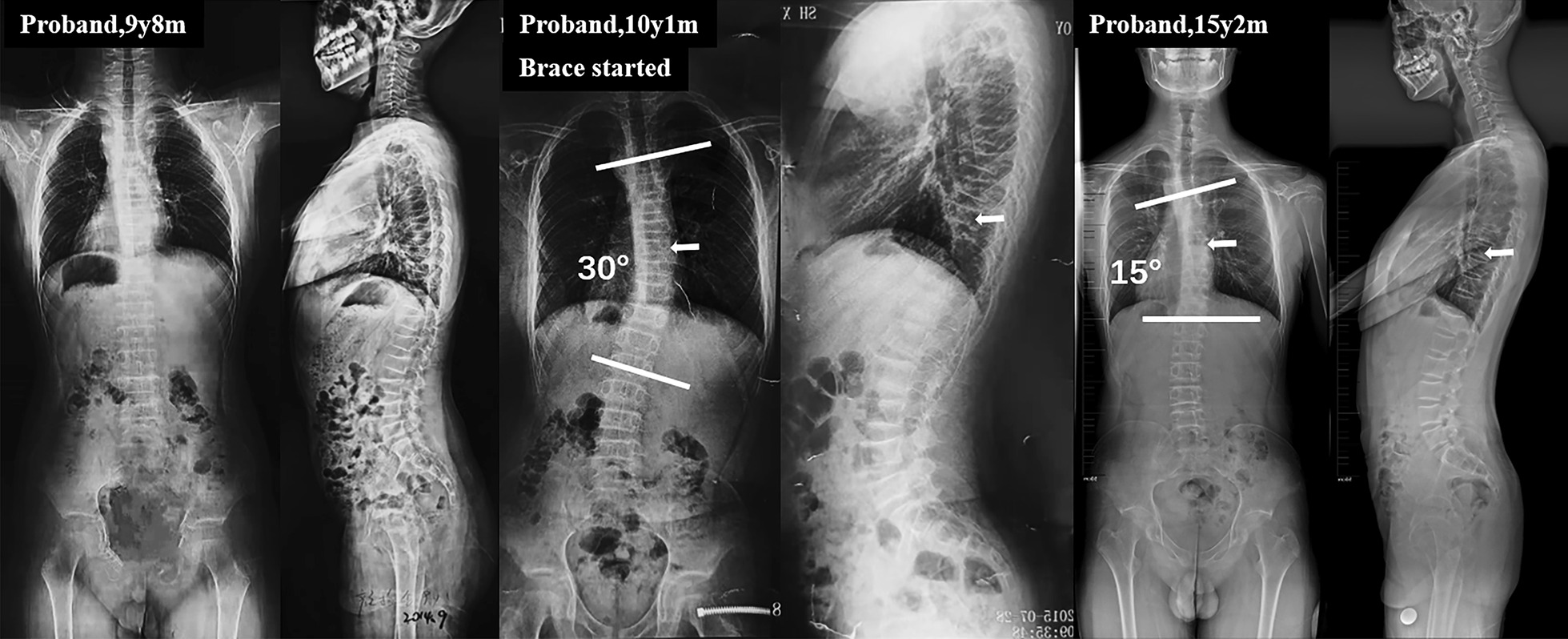
Standing anteroposterior and lateral spinal radiographs of the proband. **a** Spinal radiographs showed lumbar compression fracture. **b** The proband exhibits the phenotype of scoliosis 3 months after lumbar compression fracture. **c** The cobb angle decreased to 15° and the proband reached skeletally mature after 5 years of brace treatment. White arrows indicated the apical vertebra

The proband was a 16-year-old boy who was found to have low BMD and have sustained three low-energy fractures. He suffered from thoracic compression fracture at the age of 4 years, tibial fracture at 7 years old and lumbar compression fracture at nine. Of note, unlike other patients with early-onset osteoporosis caused by pathological mutation of PLS3 genes, the proband exhibited the phenotype of scoliosis at 10 years old (Risser sign 0). The Cobb angle of the right thoracic curve (T5-L2) was 30°, the thoracic kyphosis (T5-T12) was 41° and the lumbar lordosis (L1-S1) was 39°. He was then prescribed with brace treatment for 16 h per day. After 5 years of bracing, the proband has reached skeletal mature (risser 4) and the curve angle was decreased by 15° (Fig. 2). The younger brother of the proband, a 5-year-old boy, was also found to have an early osteoporotic fracture at 4 years of age, without scoliosis yet. Their parents were non-consanguineous, asymptomatic and reported no history of early-onset osteoporosis.

On DXA scan, as shown in Table 1, the proband had low BMD at the region of the lumbar spine (LS, Z-score − 3.3), femoral neck (FN, Z-score − 4.5) and total hip (TP, Z-score − 4.5). After 6 months of treatment with bisphosphonates, the LS BMD z-score of proband increased from − 3.3 to − 3.0. The brother of the proband also had lower LS BMD (Z-score − 1.3), FN BMD (Z-score − 3.2) and TP BMD (Z-score − 2.4). They have vitamin D deficiency as indicated by decreased concentration of 25OHD and increased cross-linked C-telopeptide of type I collagen (β-CTX) compared with age-matched normal values. Their mother, a 40-year-old female, was found to have osteopenia, with decreased LS BMD (Z-score − 1.2), FN BMD (Z-score − 1.6) and TP BMD (Z-score − 2.4). No characteristic extraskeletal manifestations of osteogenesis imperfecta, such as hearing loss, dentinogenesis imperfecta, blue sclera or facial dysmorphism were noted in all the members.

**Table 1:** Clinical characteristics of the family members

	Proband	Brother	Mother	Reference
Age at first visit (years)	16	5	40	–
Height (cm)	178	119	166	–
Weight (kg)	64	24	54	–
LS BMD (g/cm^2^)	0.650	0.505	0.974	–
LS BMD Z-score	− 3.3	− 1.3	− 1.2	–
FN BMD (g/cm^2^)	0.475	0.441	0.736	–
FN BMD Z-score	− 4.5	− 3.2	− 1.6	–
TH BMD (g/cm^2^)	0.435	0.432	0.657	–
TH BMD Z-score	− 4.5	− 2.4	− 2.4	–
Age of first fracture	4 years	4 years	–	–
Blue sclerae	No	No	–	–
Joint Hyperlaxity	No	No	–	–
Ca (mmol/L)	2.48	2.56	–	2.25–2.75
P (mmol/L)	1.42	1.33	–	0.96–1.62
25OHD (ng/ml)	19.22	25.3	–	30–50
PTH (pg/ml)	6.60	5.4	–	1.96–9.33
β-CTX (ng/ml)	1.02	1.14	–	0.26–0.512

*BMD* bone mineral density, *LS* lumbar spine, *FN* femoral neck, *TH* total hip, *Ca* calcium, *P* phosphorus, *25OHD* 25-hydroxyvitamin D, *PTH* parathyroid hormone, *β-CTX* cross-linked C-telopeptide of type I collagen

### Genetic analysis

A novel splicing mutation, c.892-2A > G, in the intron 8 of PLS3 was identified in the proband. Sanger sequencing further confirmed that the brothers were hemizygous for the mutation, their mother was typically a heterozygous carrier while the mutation was absent in their healthy father (Fig. 1b). This mutation in PLS3 was not reported in the 1000 Genomes, ExAC, PubMed and ESP6500 databases and was predicted to affect mRNA splicing by multiple splice-site prediction tools.

To unveil the effect of the mutation, we performed RT-PCR and sanger sequencing, and identified that c.892-2A > G mutation led to the skipping of exon 9 in the affected brothers, while the mother showed skewed X‐inactivation (Fig. 1c, d). The skipping of exon 9 of PLS3 leads to in-frame deletion of 32 amino acids (p.298-329del) which reside in the highly conserved actin-binding domain (Fig. 1e). This mutation was expected to produce a truncated protein with actin-binding structure being disrupted[11].

### Genotype–phenotype correlation analysis

**Fig. 3:**
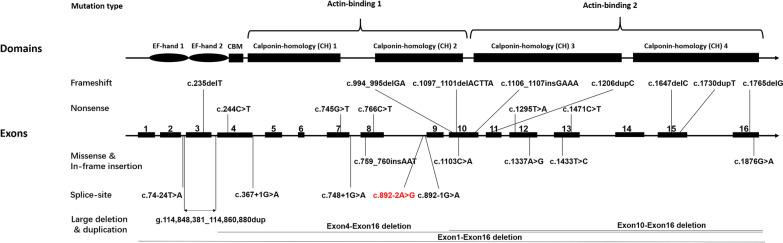
Schematic representation of the previously reported deleterious mutations in PLS3 gene and corresponding protein domains. Detained information regarding location, clinical characteristics of male patients and reference were listed in Additional file 1: Table S1. CBM calmodulin-binding motif

**Fig. 4:**
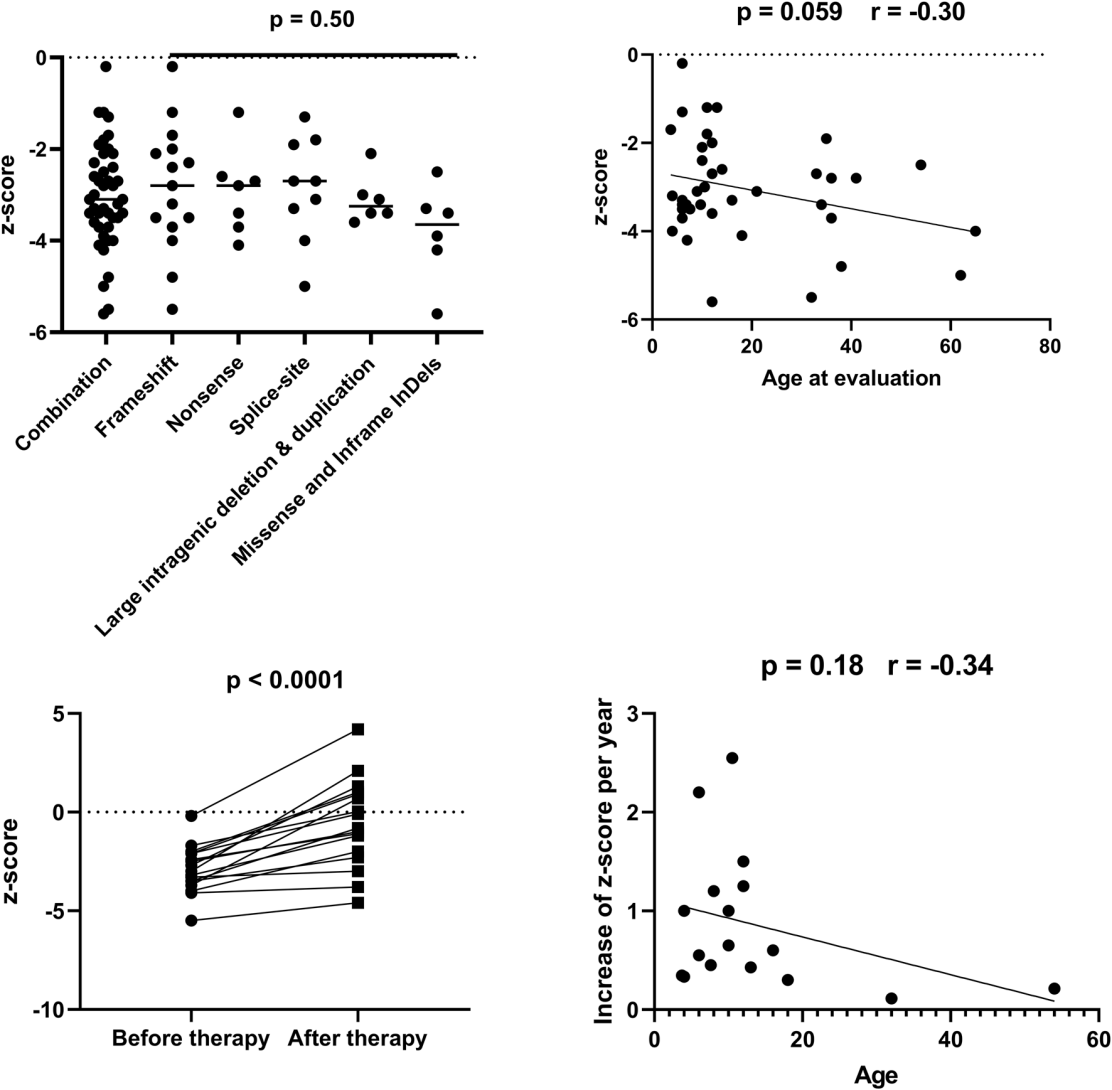
Analysis of the genotypes and phenotypes of the male patients. **a** Comparison of BMD z-score between different types of mutations. **b** The correlation between BMD z-score and age at evaluation. **c** BMD z-score before and after bisphosphonates therapy in affected males. **d** The association of the increase of BMD z-score per year and the age in the affected males underwent bisphosphonates therapy

A total of 43 males from 26 families harboring either a frameshift (N = 15), nonsense (N = 7), splice-site (N = 9), large intragenic deletion/duplication (N = 6) or missense/in-frame insertion (N = 6) were included. The detailed clinical and genetic information of the patients were listed in the Additional file 1: Table S1 and Fig. 3. Patients with PLS3 mutations exhibit variable phenotypes, while no significant association of BMD z-score with specific types of mutation was found (*p* = 0.50) (Fig. 4a). Based on the clinical information of the patients, we found a marginally significant negative correlation between age at evaluation and BMD z-score (*p* = 0.059, r = 0.30) (Fig. 4b). The conditions of osteoporosis in all patients were improved after the treatment with bisphosphonates, with mean BMD z-score increasing from − 2.9 to − 0.57 (*p* < 0.0001) (Fig. 4c). Although the correlation between age and efficacy of bisphosphonates therapy failed to reach significance, there is a trend towards less effective in older patients (*p* = 0.18, r = 0.34), especially in those aged more than 18 years (Fig. 4d).

### PLS3 levels and bone mineral density

**Fig. 5:**
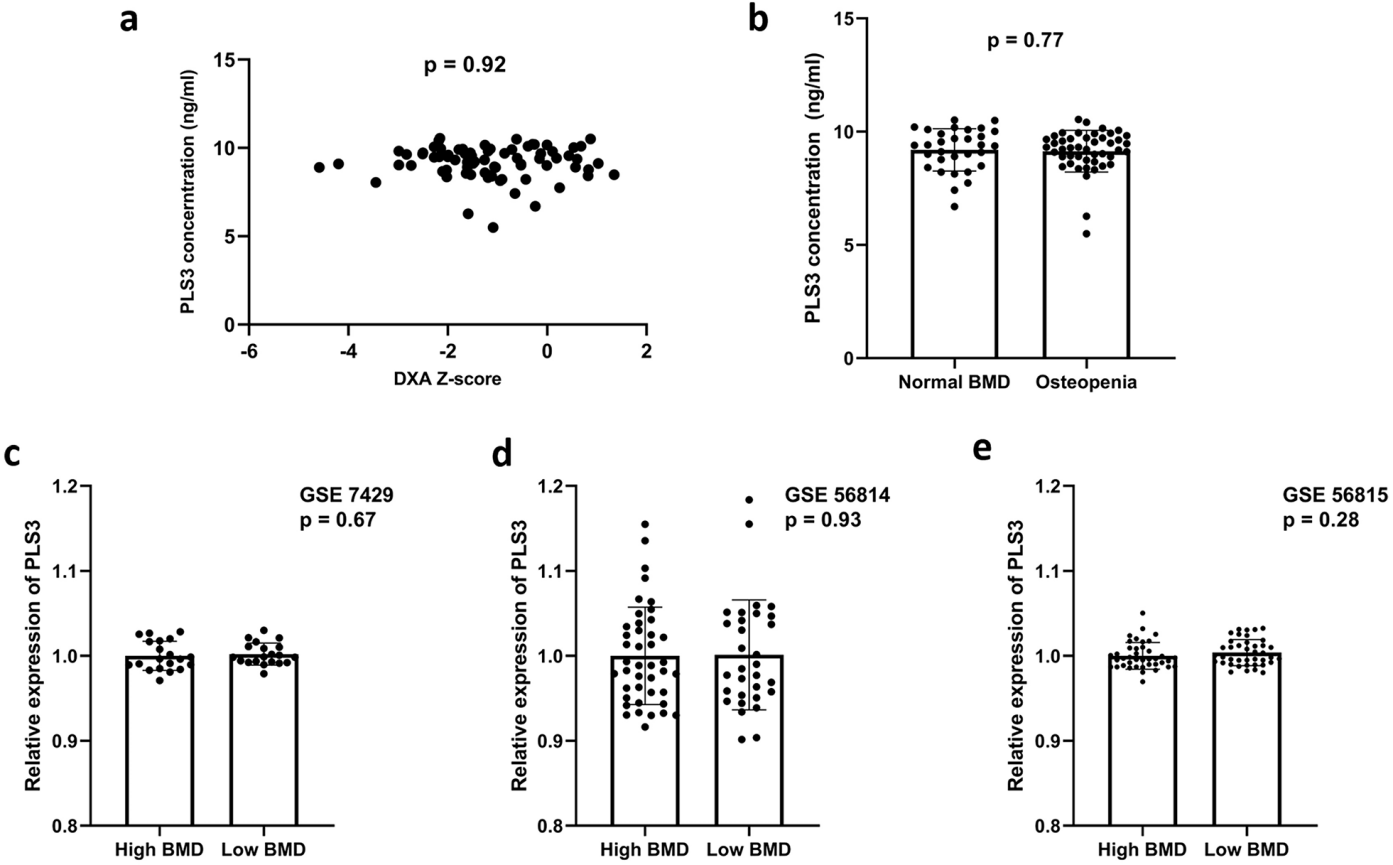
Relationship between PLS3 levels and bone mineral density. **a–b** Association of PLS3 concentration by ELISA in adolescents. **c–e** Association of PLS expression levels with bone mineral density in adults based on public datasets GSE 7429, GSE 56814 and GSE 56815

Despite mounting evidence supporting the deficiency of PLS3 caused by pathogenic mutations will result in the phenotype of osteoporosis, whether decreased PLS3 level was also involved in the occurrence of osteoporosis in general population remains unknown. To illuminate the potential biological function of PLS3, the relationship of PLS3 levels and bone mineral density in adolescents and adults was investigated. The concentration of PLS3 was comparable between osteopenia group (BMD z-score < − 1) and normal BMD group (9.2 ± 0.94 vs. 9.1 ± 0.92 ng/ml, *p* = 0.77) (Fig. 5b), and there is no significant correlation between PLS3 concentration and BMD (r = − 0.01, *p* = 0.92) in adolescents (Fig. 5a). We also did not find remarkable difference regarding PLS3 expression level between adults with high BMD and low BMD based on the datasets of GSE7429 (*p* = 0.67), GSE56814 (*p* = 0.93) or GSE56815 (*p* = 0.28) (Fig. 5c–e).

## Discussion

In the present study, we identified a novel pathogenic splicing mutation (c.892-2A > G) causing the phenotypes of early-onset osteoporotic fractures and scoliosis. The splicing mutation led to the skipping of exon 9 of *PLS3* during RNA splicing, which, in turn, produced a truncated PLS3 protein containing 32-amino acids in-frame deletion (p.298-329del). We firstly reported that scoliosis can be presented in the patients with *PLS3*-related early-onset osteoporosis, and brace treatment was effective for the controlling of curve progression and bone remodeling . Genotype–phenotype correlations, as well as association between osteoporosis and PLS3 levels in individuals without pathogenic mutations, were also investigated in the present study (Fig. 5).

To data, a total of 26 unique *PLS3* mutations have been reported to be responsible for early-onset osteoporosis in males. Most of the variants were frameshift mutations and nonsense mutations, both of which were most likely followed by nonsense-mediated mRNA decay [12]. Large intragenic deletion or duplication which can destroy gene structure and splice-site mutations that result in truncated proteins were also common. In the present study, for the first time, we compared the BMD z-score in patients with different types of mutation. Surprisingly, patients harboring mutations that caused deletion or truncated *PLS3* proteins did not exhibit more severe phenotypes when compared to those with mutated *PLS3* protein caused by missense mutations or in-frame insertion, most likely because all the types of mutations will lead to loss of function of *PLS3*. The association of age at evaluation and BMD z-score, and the effect of age on the efficiency of bisphosphonate therapy were also investigated. Our results revealed a marginally significant negative correlation between BMD z-score and age, and a trend that younger children can benefit more from the bisphosphonate therapy, possibly due to the rapid acquirement of bone mass at the age stage [13]. Considering the favorable outcomes in patients treated with bisphosphonates, our findings highlight the importance of early diagnosis and early intervention with bisphosphonates.

With early-onset osteoporosis and recurrent low-impact fractures being the clinical hallmarks of the disease, extraskeletal features, such as joint hypermobility, blue sclerae, and hearing loss can also be manifested in rare cases [3]. *PLS3* was reported to be an important cross-disease genetic modifier in multiple neuromuscular diseases [14]. Patients harboring *PLS3* mutations can also present waddling gait and clumsy gait. Change of spinal structures was a crucial part of the phenotypic spectrum of the disorder. Vertebral compression fractures (VCFs) or kyphosis occurs in over 75% of all the affected individuals [15]. A cross-sectional cohort study by Mäkitie et al. reveals that pathogenic variants in *PLS3* cause significant, early-onset and sex-related pathology in the axial skeleton both in males and females [15]. In this study, we firstly identified scoliosis to be a novel form of spinal changes, which may be controlled or even improved by brace treatment. Still, bisphosphonates should be prescribed simultaneously. There is a huge variation of osteoporotic phenotype among heterozygous women, and heterozygous female patients are generally less severely affected compared with hemizygous males [4]. The phenomenon can be explained by the variability in X inactivation, which is a process by which one of the copies of the X chromosome is inactivated in therian female mammals [9]. In this study, only the product from wild-type allele by RT-PCR was yielded from the mother, indicating that she has completely skewed the X-inactivation towards the deleterious splicing mutation. Further studies were required to broaden the phenotypic spectrum and generate targeted treatments.

*PLS3*, also known as BMD18 or T-plastin, is the most abundant isoform of plastins which are a family of actin-binding and bundling proteins that are conserved throughout eukaryote evolution. The amount of *PLS3* in the cells must be tightly regulated and precisely balanced. Aberrantly decreased or increased *PLS3* level was reported to be linked with the development and/or severity of cancer, bone disorders and neurodegeneration disorders [4]. The crucial role of *PLS3* in the regulation of bone mineral density has drawn attention of researchers during recent years, since pathogenic mutation or knockout of *PLS3* caused early-onset osteoporosis while overexpression of *PLS3* triggered osteoarthritis [16, 17]. Despite mounting evidence that the deficiency of normal *PLS3* in patients with pathogenic mutations leads to early-onset osteoporosis, whether abnormal *PLS3* level was associated with osteoporosis in individuals without pathogenic mutations remains to be elucidated. In the present study, for the first time, we evaluated *PLS3* levels in adolescents and adults with osteoporosis. However, there is a lack of variation of *PLS3* expression among all the subjects and no significant difference between subjects with high BMD and low BMD was observed. Lack of variation of *PLS3* levels among different individuals and normal levels of *PLS3* in osteoporotic patients without pathogenic mutations is a valuable observation for the reference of future research. Further studies were warranted to investigate the underlying mechanism of *PLS3*-related early-onset osteoporosis resulting from pathogenic mutations.

## Conclusion

Our finding expanded the genetic and phenotypic spectrum of *PLS3*-related osteoporosis and highlights the importance of early diagnosis and early intervention with bisphosphonates for the affected individuals. No alternation of serum *PLS3* levels in adolescents or adults with decreased bone mineral density was found in this study. Further functional study should be performed to unveil the biological mechanisms underlying genetic variants in *PLS3* on early-onset primary osteoporosis.

## Supplementary Information

**Additional file 1.** Supplementary Table 1. Clinical findings of male patients with X-linked early-onset osteoporosis caused by PLS3 pathogenic mutations.

## Data Availability

The data that supports the findings of this study are available on request from the corresponding author.

## Abbreviations

BMD: Bone mineral density
FN: Femoral neck
LS: Lumbar spine
PLS3: Plastin3
TP: Total hip
VCF: Vertebral compression fracture
WES: Whole exome sequencing
