# Supplementary material for: Identification of a novel splicing mutation and genotype–phenotype correlations in rare PLS3-related childhood-onset osteoporosis
Source: Orphanet J Rare Dis. 2022 Jun 25;17:247. doi: 10.1186/s13023-022-02380-z (PMC9233774; doi:10.1186/s13023-022-02380-z)
Supplement: Supplementary file 1 — Additional file 1. Supplementary Table 1. Clinical findings of male patients with X-linked early-onset osteoporosis caused by PLS3 pathogenic mutations. [file 13023_2022_2380_MOESM1_ESM.docx]

**Table S1. Clinical findings of male patients with X-linked early-onset osteoporosis caused by PLS3 pathogenic mutations**

| **Mutation type** | **Mutation**  **[ref]** | **Region** | **Amino acid change** | **Male patients** | **L1-L4 LS BMD Z-score (Age at evaluation)** | **Age at first fracture (years)** | **Low-energy peripheral fractures(times)** | **VCFs** | **Extraskeletal features** |
| --- | --- | --- | --- | --- | --- | --- | --- | --- | --- |
| Frameshift | c.235delT [17] | Exon3 | p.Tyr79Ilefs*6 | 1.III-2 | -5.5(32) | 2 | Yes (13) | Yes | None |
|  |  |  |  | 1.IV-1 | -1.2(13) | 8 | Yes (1) | No | None |
|  |  |  |  | 1.IV-2 | -2.1(10) | child-hood | Yes (6) | No | Acute lymphatic leukemia |
|  |  |  |  | 1.IV-3 | -3.2(4) | 4 | Yes (1) | No | Joint hypermobility |
|  |  |  |  | 1.IV-7 | -3.7(6) | 6 | Yes (17) | No | Joint hypermobility, patent ductus arteriosus and, in childhood, waddling gait |
|  |  |  |  | 1.IV-8 | -2.4(10) | child-hood | Yes (Multiple) | No | Joint hypermobility, epilepsy and, in childhood, waddling gait |
|  | c.994_995delGA [23] | Exon 10 | p.Asp332* | P1 | -3.5(7.6) | 2.5 | Yes (4) | Yes | Clumsy gait, mild spastic cerebral palsy |
|  |  |  |  | P2 | -1.7(3.7) | 2.2 | Yes (1) | Yes | None |
|  | c.1097_1101delACTTA^1^ [24] | Exon10 | p.Asn366Serfs*5 | Patient | -3.5(6) | 2.5 | Yes (NA) | Yes | Blue sclerae, joint hypermobility, hearing loss, dysmorphic facial features |
|  | c.1106_1107 insGAAA [25] | Exon10 | p.Phe369Leufs*5 | Proband | -2.0(12) | 4 | Yes (12) | Yes | Blue sclerae |
|  |  |  |  | Brother | -0.2(6) | 5 | Yes (2) | No | Blue sclerae |
|  | c.1206dupC^2^ [26] | Exon11 | p.Val403Argfs*7 | Patient9 | -2.3(NA) | 13 | Yes | NA | NA |
|  | c.1647delC [5] | Exon 15 | p.Ser550Alafs*9 | 5.II-3 | -2.8(41) | NA | Yes (10) | Yes | None |
|  | c.1730dupT [27] | Exon15 | p.Thr578Asnfs*4 | 2.III-1 | -4(4) | 2 | Yes (2) | Yes | Grey sclerae |
|  | c.1765delG [28] | Exon16 | p.Ala589Glnfs*22^3^ | Patient1 | -4.8(38) | 12 | Yes (NA) | Yes | None |
| Nonsense | c.244C > T [29] | Exon4 | p.Gln82* | III-8 | -3.7(36) | before 10 | Yes (1) | Yes | None |
|  |  |  |  | IV-5 | -2.6(14） | 7 | Non | No | None |
|  | c.745G>T [30] | Exon7 | p.Glu249* | IV-1 | -1.2(11) | 6 | Yes (2) | Yes | Blue sclera |
|  | c.766C>T^4^ [31] | Exon8 | p.Arg256* | Cohort1-Patient1 | -4.1(18) | 9 | Yes (4) | Yes | Slightly blue sclerae, slightly yellow teeth and loss of enamel, generalized joint hyperlaxity, soft skin, minor aortic valve regurgitation, and asthma |
|  | c.1295T>A [28] | Exon12 | p.Leu432* | Patient2 | -2.7(12) | 2 | Yes (NA) | Yes | NA |
|  | c.1471C>T [5] | Exon 13 | p.Gln491* | 2.III-3 | -2.8(36) | child-hood | Yes (5) | No | None |
|  |  |  | p.Gln491* | 2.III-7 | -3.4(34) | 7 | Yes (13) | Yes | None |
| Splice-site | c.74-24T>A^5^ [22] | Intron2 | p.Asp25Alafs*17 | II-3 | -5(62) | 7 | Yes (3) | Yes | None |
|  |  |  |  | II-4 | -4(65) | 33 | Yes (4) | Yes | None |
|  |  |  |  | IV-1 | -1.9(35) | 21 | Yes (1) | Yes | None |
|  |  |  |  | IV-2 | -2.7(33) | 7 | Yes (10) | Yes | None |
|  |  |  |  | IV-6 | -2.7(12) | 8 | Yes (4) | Yes | None |
|  |  |  |  | IV-7 | -3.1(9) | 8 | Yes (2) | Yes | None |
|  | c.748+1G>A [5] | exon 7 | NA | 3.II-1 | NA | NA | Yes (Multiple) | Yes | Alcohol abuse, esophageal carcinoma |
|  | c.892-1G > A [32] | Intron8 | NA | P4 | -1.8(11) | 4 | Yes (5) | No | Joint hyperextension |
|  | c.892-2A>G (This study) | Intron8 | NA | Proband | -3.3(16) | 4 | Yes (1) | Yes | None |
|  |  |  |  | Brother | -1.3(6) | 4 | Yes (1) | No | None |
| Large intragenic deletion & duplication | 3.411-MB deletion [27] | Entire gene | NA | 1.III-1 | -2.1(10) | 4 | Yes (4) | Yes | History of complex partial epilepsy and a learning disability, café-au-lait macules over his right chest and groin |
|  | E4-E16 del [21] | Exon4-Exon16 | NA | F1.1 | -3.4(9.7) | NA | No | Yes | Waddling gait, facial dysmorphism, asthma, myopia, skew foot deformity and had leg length discrepancy |
|  |  |  |  | F1.2 | -3.4(6.8) | NA | No | Yes | Waddling gait, facial dysmorphism, small joint laxity, opalescent teeth |
|  | E1-E16 del [21] | Exon1-Exon16 | NA | F2.1 | -3.6(12) | 4 | Yes (7) | Yes | None |
|  | E10-E16 del [33] | Exon10-Exon16 | NA | FM-1 | -3(10.5) | 2 | None | Yes | None |
|  | g.114,848,381_114,860,880dup  [34] | Intron 2–3 | NA | Patient | -3.1(21) | child-hood | Yes (10) | Yes | NA |
|  |  |  |  | Brother | low | NA | Yes (3) | Yes | NA |
| Missense | c.1103C>A [35] | Exon10 | p.Ala368Asp | Patient 2 | -5.6(12) | NA | Yes (NA) | NA | Facial dysmorphisms, blue sclerae and small joint laxity |
|  |  |  |  | Patient 3 | -4.2(7) | NA | Yes (1) | NA | Mild developmental delay, attention deficit hyperactivity disorder, deafness, inguinal/umbilical hernia |
|  | c.1433T>C [23] | Exon13 | p.Leu478Pro | P3 | -3.4(6) | 5 | Yes (2) | Yes | None |
|  |  |  |  | P4 | -3.3(6) | 4.8 | Yes (1) | Yes | None |
|  | c.1876G>A^6^ [26] | Exon16 | p.Gly626Arg | Patient10 | -3.9(NA) | 18 | Yes | NA | NA |
| In-frame Insertion | c.759_760insAAT [5] | Exon 8 | p.Ala253_Leu254insAsn | 4.II-1 | -2.5(54) | Adult-hood | Yes (1) | Yes | None |

All the above mutations were modified following HGMD Professional (NM_005032.7 (GRCh38)), and deviations are provided in the footnotes.

1In [24]: c.1096_1100delAACTT

2,6In [26] PLS3 isoform with 18 exons (NG_012518) was used

3In [28]: p.Ala589fs

4In [31]: c.1424A > G

5In [22]: c.73-24 T > A
